# The Etiology, Clinical Type, and Short Outcome of Seizures in NewbornsHospitalized in Besat Hospital/Hamadan/ Iran

**Published:** 2014

**Authors:** Mohammad Kazem SABZEHEI, Behnaz BASIRI, Hassan BAZMAMOUN

**Affiliations:** 1 Neonatologist, Department of Pediatrics, Besat Hospital, Hamadan University of Medical Sciences, Hamadan, Iran

**Keywords:** Neonatal seizure, Clinical type, Etiology, Neuroimaging, Outcome

## Abstract

**Objective:**

Seizures in neonates are very different from those of older children and adults. The aim of this study was to determine the etiology, clinical presentation, and outcome of seizures in hospitalized neonates of Besat Hospital, Hamadan, Iran.

**Material & Methods:**

In this retrospective study, we evaluated all neonates with seizures (aged 0-28 days) admitted to the Besat hospital, Hamadan, over a period of three years from September 2008 to September 2011. The data were obtained from hospital records and analyzed using SPSS 12.

**Results:**

Seizures were reported in 102/1112 (9.1%) neonatal admissions. Among neonates with seizures, 57% were male and 23.5% were preterm. The mean birth weight was 2936±677 grams and the mean gestational age was 37.60±1.94 weeks. 16.7% of them were LBW and 2.9% VLBW. In terms of seizure type, subtle seizures were observed in 38.2%, tonic in 29.4%, clonic in 26.4%, and myoclonic in 5.9% of cases. The main diagnosis in neonates with seizures included hypoxic-ischemic encephalopathy (HIE) (34.3%), infections (24.4%), intracranial hemorrhage (6.9%), hypoglycemia (5.9%), hypocalcemia (2.9%), inborn error of metabolism (1%), and unknown cause (24.5%). The mortality rate was 14.7%.

**Conclusion:**

Neonatal seizures indicate a significant underlying disease. HIE was the most common cause of neonatal seizures in our study. Therefore, efforts should be made to improve care during childbirth.

## Introduction

The first month of life is a time of increased risk of seizures (1), which are the most common symptom of neurological dysfunction in newborns (2). It is defined as paroxysmal brain dysfunction, and manifested as abnormal motor, autonomic, and behavioral activity (3).

The incidence of seizures is as high as 10-25% in a neonatal intensive care unit, where 15% will die and 35- 40% will have significant neurological deficit (3).

There are four recognizable clinical seizure types, including subtle, clonic, tonic, and myoclonic. Each one can be focal, multifocal, and generalized (4). The most common cause of neonatal seizures is hypoxic-ischemic encephalopathy (HIE), which accounts for approximately 50% of the causes of neonatal seizures (4).

Other causes include intracranial hemorrhage, intracranial infections, metabolic disorders, CNS malformations, birth trauma, drug withdrawal, and less frequent metabolic disorder such as inborn error of metabolism (5). Seizures are one of the immediate neonatal emergencies, where diagnostic and therapeutic plans are necessary because delay in therapy often results in poor neurological outcome (3). This study was conducted to determine the incidence, etiology, clinical type, and outcome of seizures in

newborns hospitalized in neonatal intensive care.

## Material & Methods

This retrospective cross-sectional study was carried out in the neonatal intensive care unit of Besat hospital of Hamadan University of Medical Sciences, over a period of three years from September 2008 to September 2011.

All infants<28 days of age who presented with seizures were included in this study.

The diagnosis of neonatal seizures based on clinical observation, and accurate description of the type of seizures according to the classification of Volpe, was as follows:

a) Subtle seizure: when neonate had jerking of eye, blinking or fluttering of eyelid, chewing, sucking or smacking orobuccal movement, swimming or pedaling movement of arms and legs, or apneic attack; 

b) Clonic seizure: when the neonate had rhythmic, repetitive shaking of isolated parts of the body;

c) Tonic seizure: when the neonate had stiffening of a part of the body;

d) Myoclonic seizure: when the neonate had sudden jerking motion. 

Seizure etiology diagnosis was based on positive clinical data, laboratory findings, and/or imaging studies (ultra sonography, CT scan, or MRI).

The Diagnosis of HIE was based on history, physical examination, Apgar score, arterial blood gas, and neuroimaging. In this study, the following criteria defined by the American Academy of Pediatrics and the American College of Obstetricians and Gynecologists were used for the diagnosis of birth asphyxia:

1) Profound metabolic or mixed acidemia (pH<7.00) in a sample of umbilical arterial blood; 2) Apgar score of 0-3>5 minute after birth; 3) Neonatal encephalopathy, such as seizures, coma, or hypotonic; 4) Multiple organ involvement (kidney, lungs, liver, heart, intestines). 

Diagnosis of neonatal infection was based on the clinical manifestation, sepsis workup, and positive blood culture. Bacterial meningitis was confirmed by positive CSF culture. Metabolic disturbances were considered as hypoglycemia (BS<40 mg/dl during the first 24 hours and <45 mg/dL after 24 hours of the birth), hypocalcemia (total serum Ca<8 mg/dL in full term and Ca<7 mg/dL in preterm neonates), and hyponatremia (Na<135 mg/ dL) and hypernatremia (Na>150 mg/dL). Intracranial hemorrhage was determined by brain CT or ultrasound. 

Drug withdrawal was confirmed by maternal history of drug abuse.

All data were extracted from medical records and entered in a Performa. The data statistical analysis was performed using SPSS software.

## Results

During the period of three years, 1112 infants<28 days of age were admitted to Pediatric Neonatology Division, of whom 102 (9.1%) had seizure, 58 (57%) were male, and 44 (43%) were female (male:female ratio was 1.31). Most of them (75.5%) were term, whereas only 25 (24.5%) neonates were preterm. The mean birth weight was 2936±677 grams and mean gestational age 37.60±1.94 weeks, and 16.7% were low birth weight (LBW) and 2.9% very low birth weight (VLBW). 

The mode of delivery in 54 (53%) cases was normal vaginal delivery (NVD) and in 48 (47%) were by cesarean section (CS) (Table 1).

**Table 1:** Demographic Characteristics of Study Population

Characteristic		N (%)
sex	Female	44 (43%)
	Male	58 (57%)
Birth weight	Mean	2936±677
Birth weight (groups)	≥2500	82 (80.4%)
	1501-2500	17 (16.7%)
	≤1500	3 (2.9%)
Gestational age (week) Gestational age	Mean	37.60±1.94
	Term	77 (75.5%)
	preterm	25 (24.5%)
Neonatal age at admission (day)	Mean	8.42±9.08
Mode of delivery	NVD	54 (53%)
	C/S	48 (47%)
Maternal disease	Preeclampsia	3 (2.9%)
	Chorioamnionitis	3 (2.9%)
	Abruptio placenta	1 (0.98%)
Family history of seizures	In parents	2 (1.96%)
	In siblings	2 (1.96%)
Anti-epileptic drugs	Monotherapy	77 (75.5%)
	Double therapy	20 (19.6%)
	Triple therapy	5 (4.9%)
Outcome	survive	87 (85.3%)
	expire	15 (14.7%)

We found that 51 (50%) neonates had first seizure before 72 hours of age (early-onset) and the rest had onset after 72 hour of age (late-onset) (table 2).

In terms of clinical type, subtle seizure was the most common type, which was seen in 39 (38.2%) neonates, followed by tonic in 30 (29.4%), clonic in 27 (26.4%), and myoclonic in 6 (5.9%) (Table 3). Among the various etiological factors of seizure, HIE was the most common. Thirty-five (34.3%) out of the 102 neonates had HIE and 25 (24.5%) had no obvious clinical cause. Septicemia alone, bacterial meningitis, intracranial hemorrhage, hypoglycemia, hypocalcemia, and inborn error of metabolism (MSUD) each accounted for, respectively, 21.6%, 2.9%, 6.9%, 5.9%, 2.9%, and 1% of seizures during admission (Table 4). 

Brain CT scan was performed in 49 (48%) neonates with seizures whose results showed HIP encephalopathy (N=28), intracranial hemorrhage (N=7), and normal finding (N=14). The mortality rate was 14.7% (N=15). 

The most common etiology of death was HIE in 46.6% (7/15) of the neonates.

## Discussion

Neonatal seizures are an important cause of neonatal morbidity and mortality. In our study, we found that the incidence of seizures in hospitalized neonates was 9.1%, which is comparable to finding of Mwaniki et al., Sheth et al, and Saliba et al (1,6,7), and lower than the results of Udani’s study (12).

**Table 2:** Age Distribution of Neonatal Seizure

Age	N	%
<24 hour	24	23.5
24-72	27	26.5
>72 hour	81	50
Total	102	100

**Table 3:** Different Type of Seizure

Type	N	%
Subtle	39	38.2
Tonic	30	29.4
Clonic	27	26.4
Mypclonic	6	5.9

**Table 4:** Etiological Factors in Relation to Outcome

Etiology	(N) %	Outcome		p-value
		survive	Expire	
Hypoxic-ischemic	35 (34.3)	28	7	0.432
encephalopathy				
Infections				
Sepsis	22 (21.6)	19	3	0.532
Meningitis	3 (2.9)	2	1	0.319
ICH	7 (6.9)	7	0	0.217
Hypoglycemia	6 (5.9)	6	0	0.278
Hypocalcemic	3 (2.9)	3	0	0.751
IEM	1 (1)	0	1	0.69
Unknown	25 (24.5)	22	3	0.774
Total	102 (100)	87	15	

Population-based studies estimate an incidence range of 1-3.5/1000 live birth (7). The high incidence in our study reflects the frequency of seizures in establishing intensive care units and it is acceptable because the incidence is 10-25% in the NICU (3).

The age distribution of neonatal seizure was observed in our study, and it was found that 50% of the neonates had neonatal seizures of early onset (before 72 hours). 

This high incidence in our study may be due to the fact that HIE was the most dominant factor contributing to neonatal seizures (34.3%), which is in accordance with Digra et al, and Tekgul et al’s observations (2, 11). Fiaz et al. and Ross et al., respectively, found that 59.6% and 81% of neonates had early onset seizure (3, 13). 

In our study, the most common type of seizure was subtle (38.2%), followed by tonic (29.4%), clonic (26.4%), and myoclonic (5.9%). Similar results were reported by moayedi et al. and Tekgul et al (8, 11). In contrast to these results, Fiaz et al. studied 101 neonates with seizures, of whom 39.6% had both tonic and clonic seizures (3).

Among the etiological factors, HIE (34.3%) was the most common cause. This finding is comparable to studies by Moayedi et al, Ronen, Arpino et al, and Nunes et al (8, 10, 17).

In our study, there were infections in 24.5% of cases. Takande et al. and Fiaz et al. also reported infections in 28.2% and 28.7% of neonates, respectively (14,3), which are similar to our finding, but a study by Legido et al. showed that 17.2% of neonates had infections. This difference was due to the high incidence of infection in our center.

In the present study intracranial hemorrhage was observed in 6.9% of patients, which is similar to Malik et al.’s observations (16), however, Ross et al. (13) and Fiaz et al and Taghdiri, et al. (3,18) reported higher incidence of intraventricular hemorrhage (IVH). This difference was because most of the neonates in our study were full-term, whereas intracranial hemorrhage occurs more frequently in preterm than term neonates. 

The most common metabolic disturbances were hypoglycemia and hypocalcemia, which is consistent with the observations of Fiaz et al (3). 

The mortality rate observed in our study was 14.7%, which was higher than that (9%) reported in the study by Ronen et al (9), but similar to the finding reported by moayedi et al. (13.6%) (8). This increased mortality may be due to the severity of the etiological factors in newborns with neonatal seizures. HIE and infections are the most common leading causes of mortality in our study and therefore, prevention of asphyxia and treatment of maternal infections can reduce this rate. The most common risk factor of hearing loss were hyperbilirubinemia, asphyxia,birth weight less than 1500 gr, Septicemia, convulsion, and meningitis (19).

The main limitation of this study was unavailability to obtain EEG/aEEG for all neonates with seizure at the time of our study, because EEG plays a crucial role in the evaluation of neonatal seizures.

**In conclusion, **neonatal seizures usually indicate significant underlying disease. HIE was the most common cause of neonatal seizure and mortality in our study. 

Therefore, effort should be made to improve intrapartum care. Recognition of etiological factors is helpful in prognosis and treatment. The best outcome was seen with transient metabolic abnormality.
